# Telomerase activated thymidine analogue pro-drug is a new molecule targeting hepatocellular carcinoma

**DOI:** 10.1016/j.jhep.2014.05.027

**Published:** 2014-11

**Authors:** Mirko Tarocchi, Simone Polvani, Anna Julie Peired, Giada Marroncini, Massimo Calamante, Elisabetta Ceni, Daniela Rhodes, Tommaso Mello, Giuseppe Pieraccini, Alessandro Quattrone, Claudio Luchinat, Andrea Galli

**Affiliations:** 1 Department of Experimental and Clinical Biomedical Sciences, University of Florence, Florence, Italy; 2 ProtEra S.r.l., University Scientific Campus, Sesto Fiorentino, Florence, Italy; 3 ICCOM-CNR Florence, Italy; 4 MRC Laboratory of Molecular Biology, Cambridge, UK; 5 Mass Spectrometry Centre (CISM), University of Florence, Florence, Italy; 6 Magnetic Resonance Center (CERM), University of Florence, Sesto Fiorentino, Florence, Italy; 7 Laboratory of Translational Genomics, Centre for Integrative Biology, University of Trento, Italy; 8 Department of Chemistry, University of Florence, Sesto Fiorentino, Florence, Italy; 9 Giotto Biotech S.r.l., University Scientific Campus, Sesto Fiorentino, Florence, Italy

**Keywords:** ACV, acyclovir, ACV-DP, acyclovir diphosphate, ACV-TP, acyclovir triphosphate, ACV-TP-dA, acycloguanosyl 2-deoxy-5-adenosyltriphosphate, ACV-TP-C, acycloguanosyl 5-cythydyltriphosphate, ACV-TP-dG, acycloguanosyl 2-deoxy-5-guanosyltriphosphate, ACV-TP-T, acycloguanosyl 5-thymidyltriphosphate, BrdU, bromodeoxyuridine, DN-hTERT, dominant negative human telomerase reverse transcriptase, HCC, hepatocellular carcinoma, hTERT, human telomerase reverse transcriptase, IC_50_, half maximal inhibitory concentration, IHC, immunohistochemistry, PCNA, proliferating cell nuclear antigen, Hepatocellular carcinoma, Telomerase, Acyclovir, Cancer therapy, hTERT

## Abstract

**Background & Aims:**

Hepatocellular carcinoma (HCC) is one of the most common malignancies worldwide. Although hepatectomy and transplantation have significantly improved survival, there is no effective chemotherapeutic treatment for HCC and its prognosis remains poor. Sustained activation of telomerase is essential for the growth and progression of HCC, suggesting that telomerase is a rational target for HCC therapy. Therefore, we developed a thymidine analogue pro-drug, acycloguanosyl-5′-thymidyltriphosphate (ACV-TP-T), which is specifically activated by telomerase in HCC cells and investigated its anti-tumour efficacy.

**Methods:**

First, we verified *in vitro* whether ACV-TP-T was a telomerase substrate. Second, we evaluated proliferation and apoptosis in murine (Hepa1-6) and human (Hep3B, HuH7, HepG2) hepatic cancer cells treated with ACV-TP-T. Next, we tested the *in vivo* treatment efficacy in HBV transgenic mice that spontaneously develop hepatic tumours, and in a syngeneic orthotopic murine model where HCC cells were implanted directly in the liver.

**Results:**

*In vitro* characterization provided direct evidence that the pro-drug was actively metabolized in liver cancer cells by telomerase to release the active form of acyclovir. Alterations in cell cycle and apoptosis were observed following *in vitro* treatment with ACV-TP-T. In the transgenic and orthotopic mouse models, treatment with ACV-TP-T reduced tumour growth, increased apoptosis, and reduced the proliferation of tumour cells.

**Conclusions:**

ACV-TP-T is activated by telomerase in HCC cells and releases active acyclovir that reduces proliferation and induces apoptosis in human and murine liver cancer cells. This pro-drug holds a great promise for the treatment of HCC.

## Introduction

Hepatocellular carcinoma (HCC) is the primary malignancy of hepatocytes and the most frequent solid tumour of the liver. Half a million cases occur annually in the world making it the fifth most common malignancy in men and the ninth in women [1]. Hepatocarcinogenesis is a multistep process, involving genetic and epigenetic events that accumulate during chronic liver diseases. The extent of hepatic dysfunction limits therapeutic options for HCC and the prognosis of patients with this tumour remains dismal, as the average survival from the time of diagnosis of unresectable HCC is measured in months [2]. Therefore, in this dramatic scenario, HCC is an attractive target for the identification of new chemotherapeutic agents.

The current trend in research on anti-cancer drugs is to exploit particular traits or hallmarks that are unique to cancer cells. Despite the fact that cancers display a great heterogeneity in clinical behaviour, most human tumours, including HCC, share a limited set of acquired capabilities that define the malignant state [3], [4]. Emerging insights into the biology and molecular signalling pathways of HCC cells have led to the identification of several potential new targets for intervention and the advent of numerous targeted therapies for the treatment of this otherwise lethal tumour. The recent use of Sorafenib, a multikinase inhibitor, is a step forward in the treatment of advanced HCC [5], [6], and represents the beginning of a new horizon in the molecular targeted therapy of HCC [7]. Another particularly interesting target is the unlimited replicative potential of cancer cells via the activation of a telomere maintenance mechanism, which is a key step to ensure expansive tumour growth [8].

Telomerase is a ribonucleoprotein complex containing the reverse transcriptase enzyme hTERT that adds repetitive DNA sequences to telomeres, preventing telomere shortening and consequently cell death. Telomerase activity in the adult is present only in a few proliferating cell types, including germ cells, bone marrow stem cells, and epithelial basal cells [9]. In contrast to normal cells, malignant cells from a large variety of human tumours contain active telomerase, which plays a key role in cellular immortalization [10]. In HCC, telomerase reactivation has been detected in over 80% of cases and gene expression analyses have shown that *hTERT* can be included in sets of selected genes that provide molecular signatures, used for the diagnosis and the management of hepatic nodules [11], [12]. Moreover, *hTERT* mRNA levels increase during progression of HCCs and correlate with the transition between low- and high-grade dysplastic nodules [13]. Therefore, telomere maintenance and hTERT are considered potential targets for anti-cancer drug development.

The well-known antiviral agent acyclovir (ACV) is a nucleotide analogue acting as a DNA chain terminator [14], mainly used in the treatment of herpes virus infection. The high selectivity of ACV for virus-infected cells is due to the presence of viral thymidine kinases (TK) that specifically and selectively phosphorylate ACV to acyclovir monophosphate (ACV-MP). Human enzymes, guanylate monophosphate (GMP) kinase and nucleoside diphosphate (NDP) kinase, then further phosphorylate ACV-MP to di- (ACV-DP) and triphosphate (ACV-TP), and the latter is incorporated into DNA, arresting its replication [15] (Supplementary Fig. 1). On this basis, adenoviruses carrying the herpesvirus TK gene have been proposed in combination with ACV or its analogues as anti-cancer agents in suicide gene therapy. However, the use of a virus in patients raises ethical concerns, and is undermined by the risk of low and transient expression levels of the transgene [16], [17]. We previously have shown in pancreatic cancer the efficacy of acycloguanosyl 5′-thymidyltriphosphate (ACV-TP-T), an ACV-derived pro-drug that is constituted by a thymidine triphosphate attached to the hydroxyl group of ACV [18]. This evidence together with the knowledge of the important role of telomerase in HCC prompted us to test the anti-neoplastic effect of this pro-drug against liver cancer.

In this paper we now provide direct evidence from both *in vitro* analyses and *in vivo* experiments in HCC cells that ACV-TP-T is actually a telomerase substrate, which by incorporating the thymine base in DNA synthesis releases the acyclovir active form. Importantly, we find that ACV-TP-T treatment inhibits tumour growth both in *in vitro* and *in vivo* models of hepatocarcinogenesis.

## Materials and methods

Detailed information on the experimental procedures are provided in the Supplementary material and methods.

## Results

### ACV-TP-T effects on hepatocellular cancer cell lines

Based on the evidence of increased telomerase activity in HCC tissues compared to normal liver [19], human (HepG2, Hep3B, and HuH7) and mouse (Hepa1-6) hepatocellular cancer cell lines were exposed to increasing concentrations of ACV-TP-T (from 0.1 to 1000 μmol/L) for 24 h. ACV-TP-T inhibited DNA synthesis in a dose-dependent manner in all tested cell lines and the calculated half maximal inhibitory concentration (IC_50_) ranged from 3 to 30 μmol/L (Table 1). Telomerase activity was also tested, and as expected, telomerase activity was present in all liver cancer cell lines analysed and its level was comparable to that of other cancer cell lines from different tissues (data not shown). Interestingly, normal cells derived from healthy human colon, 18CO, showed no presence of telomerase activity and, as expected, ACV-TP-T had no effect on viability or DNA synthesis.

**Table 1:** **Cytotoxicity (based on ATP concentration) and anti-proliferative activity (based on [^3^H] thymidine incorporation) of ACV-TP-T in four different hepatic cancer cell lines and one normal colon cell line in comparison with telomerase activity.**

### In vitro activation of ACV-TP-T by telomerase

**Fig. 1:**
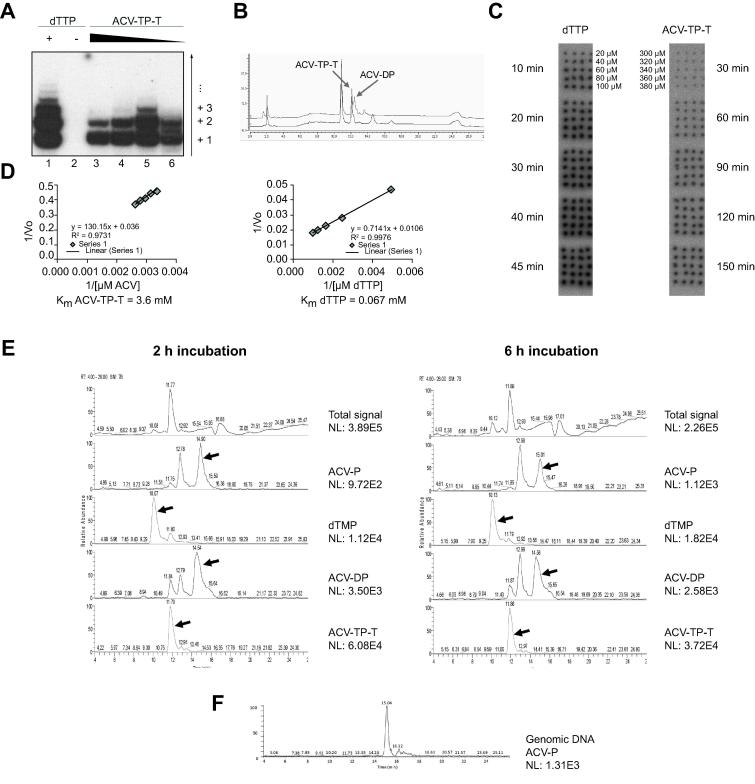
**ACV-TP-T can substitute dTTP as substrate for telomerase.** (A) DNA synthesis by telomerase was analysed on denaturing PAGE. Lane 1: Partially purified telomerase was incubated for 6 h at 37 °C in the presence of 0.25 μM telomeric (TTAGGG) 3 primer, 1 mM dTTP, 1 mM dATP, 0.1 mM dGTP, and 0.17 μM dGTP [α-32P]; lane 2: reaction conditions as in lane 1 but without dTTP; lanes 3–6: conditions as in lane 1 but dTTP was substituted with 2 mM, 1 mM, 0.1 mM or 0.02 mM ACV-TP-dT respectively. (B) Chromatographic analysis of telomerase reaction products under conditions as in (A), lane 3 (upper line); as a control, the reaction mix was incubated in the absence of telomerase (lower line). ACV-DP is not present in the medium without telomerase. In the presence of telomerase, ACV-TP-T is hydrolysed (about 30%) leading to the formation of ACV-DP. (C) Telospot time courses where super-telomerase was incubated with 300, 320, 340, 360, 380 μM of ACV-TP-dT or 20, 40, 60, 80, 100 μM dTTP. All other reaction components were kept as in (A). Reactions with ACV-TP-T were quenched after 30, 60, 90, 120, 150 min and reactions with dTTP after 10, 20, 30, 40, 45 min. 0.5 μl out of each 20 μl reaction were manually spotted 4 times on a nylon membrane, which was then probed with a randomly radiolabelled telomeric DNA. Reaction products were quantified upon exposure of the membrane to a Phosphorimager. (D) Double reciprocal Lineweaver–Burk plots of initial velocities as determined from panel (C). (E) LC-MS/MS analysis of cell lysates after an incubation time of 2 h (left panel) and 6 h (right panel). The total ion current (top panel) and the ion current from the sum of two major fragment ions of each molecule of interest (lower panels) and their absolute intensities (NL) are shown. (F) LC-MS/MS analysis of genomic DNA from Hepa1-6 cells after an incubation time of 6 h (panel E); DNA was purified and extensively degraded.

To demonstrate that ACV-TP-T is a telomerase substrate (Supplementary Fig. 1) and that its effect on cancer cell lines depends on its enzymatic activation of the pro-drug, we performed a direct telomerase activity assay using partly purified telomerase from a super-telomerase cell line [20] and decreasing concentrations of ACV-TP-T. Fig. 1A clearly shows that ACV-TP-T is used by telomerase as a substrate in place of dTTP. To investigate, whether telomere synthesis and incorporation of ACV-TP-T by the telomerase releases ACV-DP, the telomere synthesis reaction was analysed by chromatography. The analysis of the telomerase reaction after 6 h of incubation shows that whereas the concentration of ACV-TP-T decreased by about 30%, a new peak representing the formation of about 30% ACV-DP was formed (Fig. 1B). No other products, such as ACV-TP, ACV-MP, or mono-, di- or tri-phosphorylated dT, were found in appreciable quantities. Taken together, these results confirm that telomerase functions as an activator of the pro-drug, through telomerase-dependent DNA synthesis with a concomitant release of ACV-DP.

Next, we determined the telomerase K_m_ values for ACV-TP-T and dTTP. We estimated a K_m_ value for ACV-TP-T of 3.6 mM, which is approximately 50 times higher than the K_m_ of the reaction with dTTP (Fig. 1C and D). As the physiological concentration of dTTP is in the low micromolar range [21], achieving the optimal high micromolar values of the ACV-TP-T concentration *in vivo* would ensure sufficient selection of the pro-drug by the telomerase.

### HCC cells metabolize ACV-TP-T in vitro

To demonstrate that the activation of the pro-drug occurs in hepatocellular cancer cells, Hepa1-6 cells were incubated for 2 h or 6 h with ACV-TP-T (1 mmol/L). Intracellular phosphorylated metabolites were characterized by LC-MS/MS (Fig. 1E). When cells were treated for 2 h, analysis of the cellular extract showed the presence of peaks corresponding to the pro-drug and its metabolite ACV-DP. After 6 h, the concentration of ACV-TP-T was decreased in the cells (−39%) as well as in the culture medium (−14%). Interestingly, ACV-P and dTMP were increased but the ACV active metabolite, ACV-DP, was reduced, suggesting that it was actively incorporated into the DNA after further phosphorylation. Furthermore, after genomic DNA purification and degradation, we were able to identify ACV-P in the DNA sample (Fig. 1F). These results demonstrate the ability of ACV-TP-T to enter hepatoma cells and that the pro-drug is actively metabolized inside the cells releasing the active species of ACV.

### hTERT modulation influences HCC cell susceptibility to ACV-TP-T

To confirm the role of telomerase in ACV-TP-T activation and on the correlated effect on cell growth, we modulated telomerase activity in HCC cells by transfection with wild type (hTERT-WT) or dominant negative (hTERT-DN) hTERT expression vectors. As shown in Supplementary Fig. 2, hTERT-DN overexpression in Hepa1-6 and HepG2 cells reduces telomerase activity (43% ± 3 SEM and 66% ± 2 SEM, respectively) and in parallel increases the IC_50_, indicating a lower susceptibility to the drug. On the contrary, increasing telomerase activity by hTERT-WT overexpression in the same cells brings a reduction of ACV-TP-T IC_50_ (37% ± 2 SEM and 89% ± 12 SEM, respectively). Differently, in Hep3B cells that have a high telomerase basal activity (Table 1), hTERT–WT transfection had a negligible effect on the susceptibility to ACV-TP-T, whereas hTERT-DN transfection significantly reduced telomerase activity and increased the IC_50_. On the contrary, only hTERT–WT overexpression significantly influenced the susceptibility to ACV-TP-T in HuH7 transfected cells.

Similar experiments of telomerase modulation were done in 18CO, a normal cell line with no telomerase activity; the overexpression of hTERT-WT in this cell line was not able to reduce the IC_50_ (>1000 μM) and this corresponded to a lack of restored telomerase activity even if *hTERT* mRNA levels were increased (data not shown). When the cells were transfected with hTERT-hTR the telomerase activity increased to 483 relative telomerase activity (RTA) and the cells responded to ACV-TP-T with an IC_50_ of 177 μM.

Furthermore, to confirm the role of telomerase in pro-drug activation, Hepa1-6 cells were treated with other ACV derivatives in which the thymidine was replaced with different bases. In agreement with our previous results [18] the thymidine-linked acyclovir (ACV-TP-T) was the most anti-proliferative drug and the cytosine derivative (ACV-TP-C) did not inhibit proliferation, consistently with the inability of telomerase to use cytosine as substrate (Table 2). These results indicate the lack of non-specific activity of the pro-drug.

**Table 2:** ***In vitro* effects of ACV-TP-G, ACV-TP-A, ACV-TP-T and ACV-TP-C.**

The four compounds were tested on Hepa1-6 cells measuring both viability (based on ATP concentration) and proliferation (based on thymidine incorporation).

### ACV-TP-T inhibits cancer cell proliferation by blocking cell cycle and increases apoptosis

**Fig. 2:**
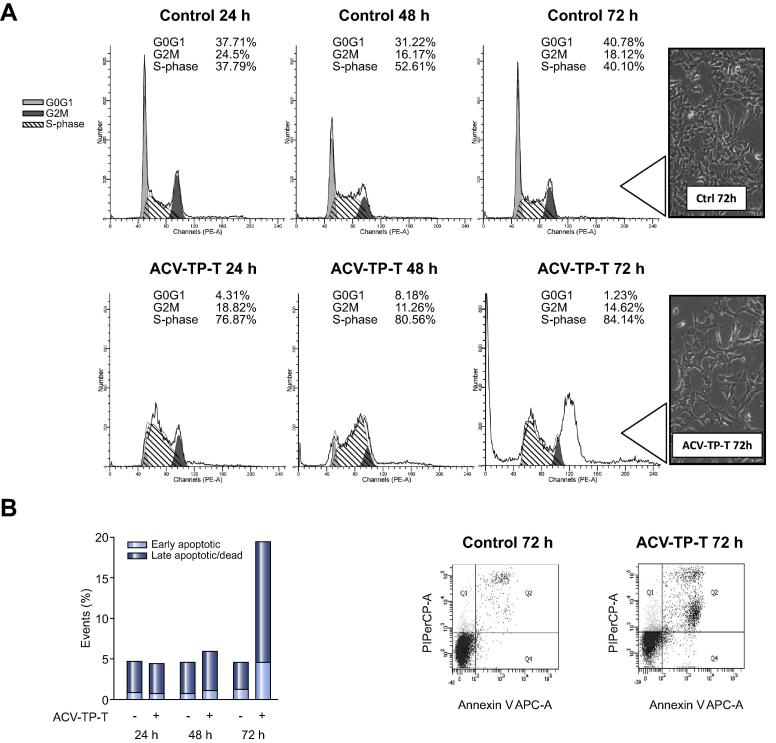
**ACV-TP-T treatment blocks HCC mouse cells in S-phase and increases cell death.** (A) Cell cycle analysis of synchronized Hepa1-6 cells treated with ACV-TP-T: data showing one representative experiment out of three with the distribution of cells in G0G1, S-phase, G2M. Inset: cell morphology at 72 h. (B) Flow cytometry analysis of Hepa1-6 cell vitality at 24 h, 48 h and 72 h (one representative experiment out of three): apoptotic and dead cells are expressed as% of total events.

To evaluate whether the evident inhibition of cell proliferation was associated with cell cycle modification, FACS analysis of Hepa1-6 cells treated with ACV-TP-T was performed at different time points. ACV-TP-T treatment at the IC_50_ concentration induced a significant arrest in S-phase that was already evident after 24 h (+37.7%) (Fig. 2A). This effect was consistently maintained at all experimental time points with an increased percentage of S-phase arrest in ACV-TP-T treated cells (*p* <0.001). Moreover, ACV-TP-T treatment was able to increase the number of apoptotic cells over time and FACS analysis by Annexin V/propidium iodide staining showed a peak at 72 h (Fig. 2B) with 18.7% ± 2.1 non-vital cells *vs.* 5.6% ± 0.9 in controls (*p* <0.001).

The capacity of ACV-TP-T to induce S-phase arrest was also confirmed in human cancer cell lines (HuH7 and Hep3B) (*p* <0.01) (Supplementary Fig. 3). Analysis of dead cells in treated and untreated HuH7 and Hep3B cells showed similar effects of ACV-TP-T in the HuH7 cell line (Supplementary Fig. 4A) (*p* <0.001), but unexpectedly Hep3B cells did not respond to ACV-TP-T exposition. Considering that Hep3B are *p53*-null cells and that this protein is a key regulator of the apoptotic process, we reintroduced wild type p53 in Hep3B cells by transfection with the pSN3-p53 vector [22]. Pro-apoptotic effects of ACV-TP-T treatment after 48 h and 72 h were significantly (*p* <0.01) increased in pSN3-p53-transfected Hep3B but not in Hep3B cells transfected with the control vector, indicating that p53 is necessary to promote cell death by ACV-TP-T (Supplementary Fig. 4B).

### ACV-TP-T induced HCC regression in HBV transgenic mice

**Fig. 3:**
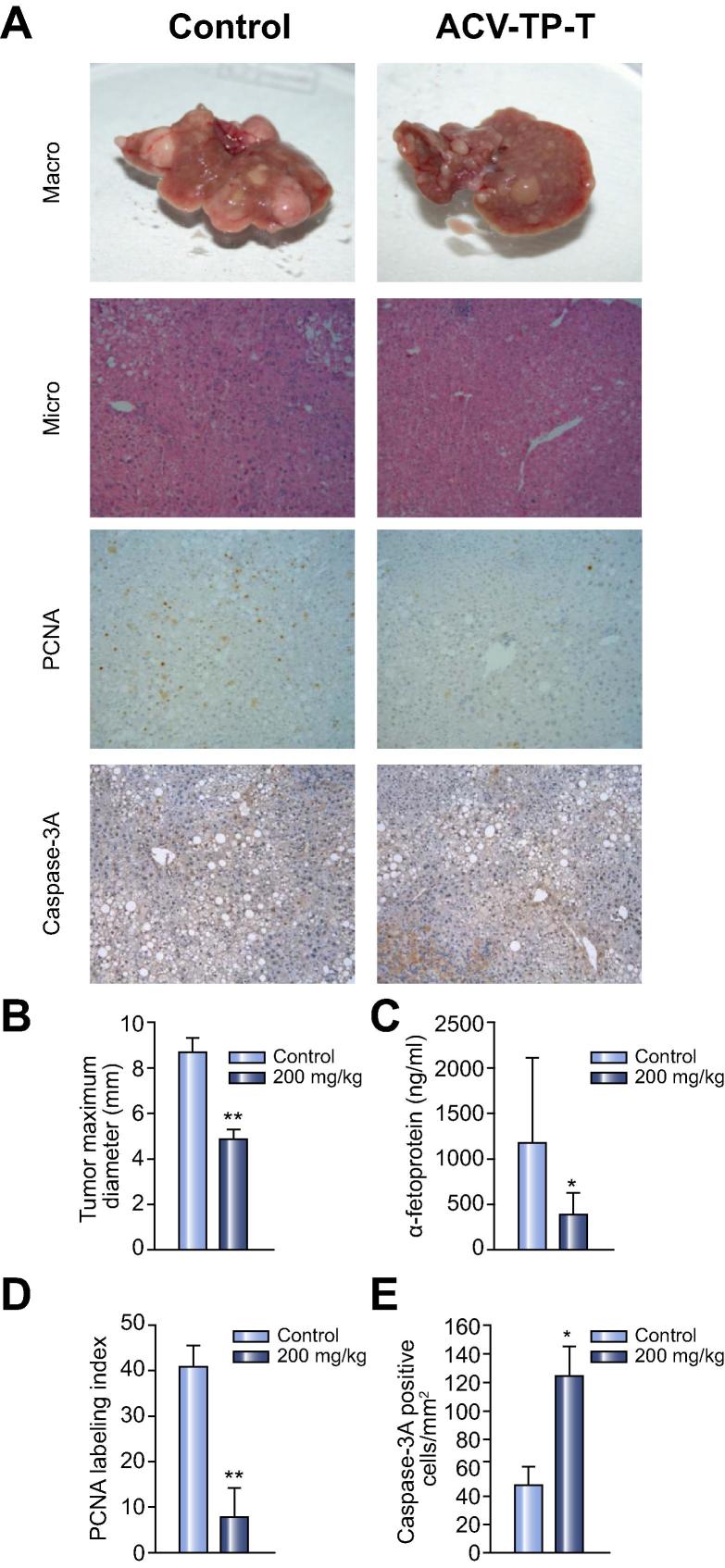
**ACV-TP-T treatment reduces neoplastic lesion size in a transgenic spontaneous hepatic tumour mouse model.** (A) Representative image of the livers for control and ACV-TP-T treated mice. Tumour cells were identified following H&E staining (100× magnification). Proliferation was assessed by PCNA antibody staining, apoptosis by activated Caspase 3 antibody staining. (B) The macroscopic tumours were identified at the time of sacrifice; the maximum diameter (cm) was measured with a caliper (^∗∗^*p* <0.01). (C) Quantification of α-fetoprotein level in the serum of the animals at the time of sacrifice (expressed as average ± SD, ^∗^*p* <0.05). (D) Quantification of PCNA labelling index (expressed as average ± SEM, ^∗∗^*p* <0.01). (E) Quantification of activated Caspase 3 positive cells (expressed as average ± SEM, ^∗^*p* <0.05).

Following the *in vitro* results we chose to examine the effect of ACV-TP-T administration in a mouse model of HBV-related hepatocarcinogenesis. Hepatocytes of HBV transgenic mice (Tg [Alb-1 HBV] Bri 44) express and accumulate the large HBsAg protein, resulting in severe chronic hepatocellular injury. This condition is always followed by the development of dysplastic hepatic lesions that progress after the 9 months to hepatocellular adenomas and carcinomas [23], [24]. Interestingly, livers of 15-month old mice that have large tumours have increased telomerase activity compared to wild type animals (Supplementary Fig. 5). ACV-TP-T or vehicle alone (control) were administered three times per week by i.p. injection to HBV transgenic mice (n = 40) for 4 weeks starting from 15 months when all animals have developed hepatocellular adenomas and hepatocellular carcinomas. ACV-TP-T-treated mice showed significantly smaller neoplastic foci compared with vehicle-treated animals. In parallel, α-fetoprotein, a marker of hepatocellular regeneration and transformation, was drastically reduced in ACV-TP-T-treated animals. The growth inhibition and the possible induction of tumour regression by ACV-TP-T correlated with the reduction of the proliferative activity and increased frequency of apoptotic liver cells as measured by immunostaining detection of PCNA and activated caspase 3, respectively (Fig. 3).

### Growth inhibition induced by ACV-TP-T evaluated in an orthotopic syngeneic mouse model of HCC

**Fig. 4:**
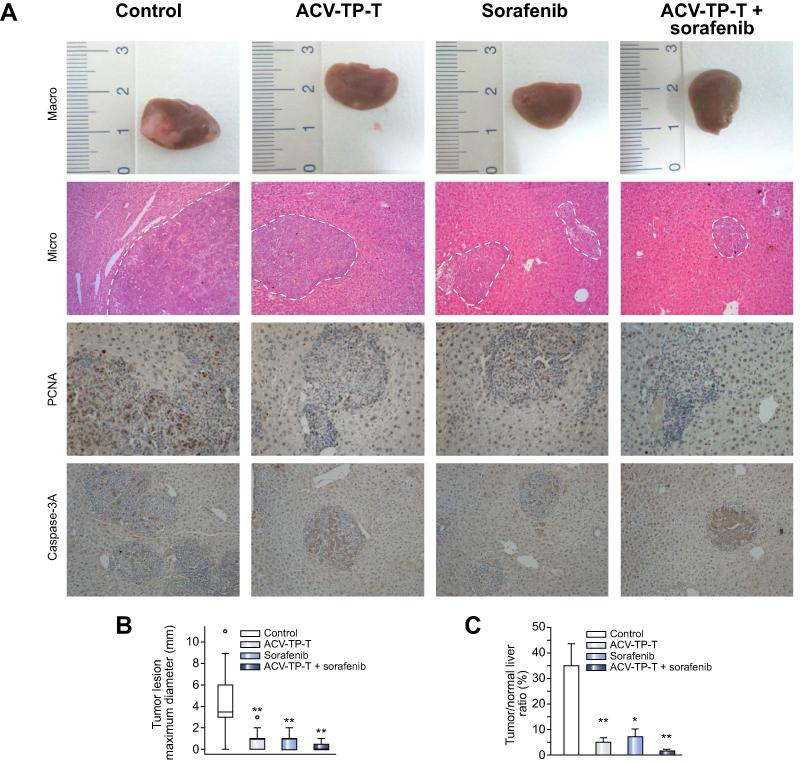
**Decreased growth of implanted Hepa1-6 cells with ACV-TP-T and/or Sorafenib treatment.** (A) Representative images of the left lobe of the livers for each group. Tumour cells were identified following H&E staining (100× magnification). Proliferation was assessed by PCNA antibody staining, apoptosis by activated Caspase 3 antibody staining. (B) The macroscopic tumours were identified at the time of sacrifice; the maximum diameter (cm) was measured with a caliper (^∗^*p* <0.05). (C) Tumour surface was estimated in the left lobe of each liver by direct measurement, and the surface ratio between the tumour and the lobe was quantified with an image analyser (BioQuant Software) (expressed as average ± SEM; ^∗∗^*p* <0.01).

We next assessed the impact of ACV-TP-T alone or in association with the multikinase inhibitor Sorafenib on tumour growth in an orthotopic mouse model. We implanted 2 × 10^6^ Hepa1-6 cells in C57Bl/6 mice (n = 40). After 2 weeks of treatment we measured macroscopically the major diameter of the formed tumours, and microscopically the total surface area of the left lobe occupied by cancer cells (Fig. 4). Interestingly, the size and relative surface area occupied by the tumours were significantly decreased in all treated animals compared to control animals whose liver left lobe was largely replaced by tumour cells. No statistical difference was found among the three treated group (ACV-TP-T alone, Sorafenib alone or in combination). During the treatment no adverse events were detected in the ACV-TP-T group, but a few deaths occurred within the first week in the Sorafenib alone (n = 1) and in the combination group (n = 3).

## Discussion

In the last decade, telomerase activation and its regulation have become a topic of intense investigation in oncology and aging-related diseases [25], [26], [27]. Activation of telomerase seems to be one of the crucial events that underlie the multigenetic process of carcinogenesis [12], [28]. In the field of HCC where the discovery of new systemic therapies is urgently needed, the well-characterized key role of telomerase in hepatocarcinogenesis makes this enzyme an attractive therapeutic target [29], [30], [31].

Several different approaches are being investigated to target telomerase. Direct inhibition of the catalytic subunit (hTERT) or its RNA template (hTER), using small molecules, antisense oligonucleotides or immunotherapy, have been proposed. Other investigators have suggested to target telomerase activity indirectly with compounds that stabilize the DNA G-quadruplex, that inhibit telomere synthesis, tankyrase or HSP90 inhibitors, thus blocking telomerase access to telomeres or inhibiting binding of telomerase-associated proteins [32], [33]. Furthermore, telomerase gene transcription in cancer was also proposed in suicide gene therapy where *Herpes simplex* virus thymidine kinase (TK), expressed under control of the telomerase promoter, was associated with ganciclovir treatment [34].

The therapeutic approach targeting telomerase presented in this study is innovative: it uses native cancer cell telomerase enzymatic activity to transform a pro-drug into an active molecule that blocks DNA synthesis necessary for cell proliferation in cancer cells. We showed that the telomerase of HCC cells metabolizes the pro-drug and releases an active form of ACV that is responsible for the inhibition of HCC cell proliferation both in *in vitro* and *in vivo* models of hepatocarcinogenesis. All HCC cell lines tested were susceptible to the anti-proliferative effect of ACV-TP-T, although the response in term of cell growth inhibition was slightly different between the different cell lines (Table 1). Our evidence suggests that susceptibility to ACV-TP-T in HCC cells might be influenced by other mechanisms. The expression of nucleoside transporters, DNA repairing systems, kinase expressions and multidrug resistant proteins might play a key role in the susceptibility and resistance of cancer cells to nucleoside analogues. These pathways have already been described in HCC [35], [36] and are currently under investigation in our laboratory. Nevertheless, we clearly demonstrated that ACV-TP-T was a telomerase substrate and that hepatocellular cancer cells took up and metabolized ACV-TP-T to release the active compound (Fig. 1E). Unexpectedly, a lower efficiency of telomere synthesis was observed at a higher concentration of ACV-TP-T in the *in vitro* enzymatic reaction (Fig 1A, compare lane 3 and 5). A possible explanation could be that in the presence of high concentrations of ACV-TP-T, telomerase might use the pro-drug from the ACV-TP side to incorporate ACV-MP in place of dG-MP, thereby interrupting telomere synthesis and therefore preventing further processing of the pro-drug [20]. In any case, these high concentrations are not relevant *in vivo*, and therefore this effect is of little concern for the possible *in vivo* use of the pro-drug. The role of telomerase in pro-drug activation was further confirmed in HCC cell cultures by the overexpression of the wild type or the dominant negative genes of hTERT, which reduced or increased ACV-TP-T efficacy in parallel with the variation of the telomerase activity levels (Supplementary Fig. 2).

In agreement with previous studies that reported an S-phase cellular arrest and induction of cell death after ACV treatment [37], [38] in infected cells, ACV-TP-T induced a block in the cell cycle, and increased the number of apoptotic cells (Fig. 2 and Supplementary Figs. 3 and 4). Besides, in Hep3B cells that are *p53*-null we were able to induce cellular arrest, but not cell death. The reintroduction of *p53* in this cell line increased the number of pro-apoptotic cells similarly to the other cell lines, indicating that p53 is necessary for the activation of the pro-apoptotic pathway. Interestingly, the treatment with ACV-TP-T in cell lines where *p53* is mutated or missing (HuH7 and Hep3B, respectively), mimicking HCC cells in which the p53 pathway is often lacking due to frequent mutations [39], was consistently able to induce cellular arrest. In consideration of the selective effects on cancer cells these data suggest that this molecule could be used alone for its cytostatic effect or in combination therapies.

As shown in our previous study [18], four week treatment with ACV-TP-T was able to reduce the growth of pancreatic adenocarcinoma sparing the healthy proliferating tissues, suggesting that this pro-drug has a highly selective activity on cancer cells that have reactivated telomerase and might be safe for the treatment of patients.

Here we also demonstrated that ACV-TP-T had anti-proliferative and pro-apoptotic effects on hepatocellular cancer cells *in vivo*: in only four weeks it was able to block the progression of the hepatic lesions and induced regression of spontaneously developed tumours in a transgenic model (Fig. 3). Furthermore, the treatment was able to reduce the growth of murine HCC cells directly implanted in the liver of wild type syngeneic animals, which in two weeks can reach over 1 cm in diameter in untreated animals (Fig. 4). This evidence strongly suggests the possible use of this molecule for the treatment of HCC, as well as for all pre-neoplastic or cancer lesions with high telomerase activity [40], [41], [42], [43], [44]. The effects obtained in the orthotopic syngeneic model also indicate that a combination of ACV-TP-T therapy targeting telomerase activity with the approved standard of care, Sorafenib, is possible and, even if it is not reaching a statistical significance against each single treatment, reduces the average diameter of nodules and the microscopical extension of cancer cell replacement of normal liver tissues (Fig. 4B and C).

In conclusion the mechanism of selective telomerase activation of the pro-drug holds great promise for the treatment of hepatocellular cancer and of other tumours characterized by high telomerase expression.

## Financial support

This work was supported by grants from the Ministero dell‘Istruzione, dell‘Universita‘e della Ricerca (FIRB RBAP10MY35_002 to A.G.), the Cassa di Risparmio di Firenze (CRF), and the FiorGen Foundation.

## Conflict of interest

The authors who have taken part in this study declared that they do not have anything to disclose regarding funding or conflict of interest with respect to this manuscript.
