# Supplementary material for: Telomerase activated thymidine analogue pro-drug is a new molecule targeting hepatocellular carcinoma
Source: J Hepatol. 2014 Nov;61(5):1064–72. doi: 10.1016/j.jhep.2014.05.027 (PMC4309885; doi:10.1016/j.jhep.2014.05.027)
Supplement: Supplementary data [file mmc1.pdf]

Supplementary data to

Telomerase activated thymidine analogue pro-drug is a new molecule targeting hepatocellular carcinoma

Mirko Tarocchi[1], Simone Polvani[1], Anna Julie Peired[1], Giada Marroncini[1], Massimo Calamante[2,3] Elisabetta Ceni[1], Daniela Rhodes[4], Tommaso Mello[1], Giuseppe Pieraccini[5], Alessandro Quattrone[6,7], Claudio Luchinat[6,8,9], Andrea Galli[1,]*

[1]Department of Experimental and Clinical Biomedical Sciences, University of Florence, Florence, Italy; [2]ProtEra S.r.l., University Scientific Campus, Sesto Fiorentino, Florence, Italy; [3]ICCOM-CNR Florence, Italy; [4]*MRC Laboratory of Molecular Biology, Cambridge, UK;* [5]Mass Spectrometry Centre (CISM) University of Florence, Florence, Italy*;* [6]Magnetic Resonance Center (CERM), University of Florence, Sesto Fiorentino, Florence, Italy; [7]Laboratory of Translational Genomics, Centre for Integrative Biology, University of Trento, Italy; [8]Department of Chemistry, University of Florence, Sesto Fiorentino, Florence, Italy; [9]Giotto Biotech S.r.l., University Scientific Campus, Sesto Fiorentino, Florence, Italy

Table of contents

Supplementary data to 1
Supplementary materials and methods 2
- Telomerase expression and purification 2
- Direct telomerase activity assay 2
- Detection of pro-drug metabolites in HCC cells 3
- Cell culture and transfection 4
- Telomerase activity assay in cell 5
- Analysis of cell proliferation and viability 5
- Cell cycle analysis 5
- Annexin V/propidium iodide staining 6
- Mouse models of Hepatocarcinogenesis 6
- Tumor evaluation 7

Immunohistochemistry (IHC) 8
Statistical analysis 8
Supplementary Figures 9
References 14

Supplementary materials and methods

Telomerase expression and purification

The telomerase complex was over-expressed in HEK293T cells transiently transfected with hTERT- and hTER-expressing plasmid DNA (pVan145; pCDNA6-ZZ-(TEV)-3xFlag-hTERT(WT) and pBS-U1-hTR) as described[1] and whole-cell lysates (WCL) were prepared as described[2]. For telomerase affinity purification, the WCL was adjusted to a protein concentration of 4 mg/ml with buffer A (20 mM HEPES-KOH pH 7.9, 2 mM $MgCl_2$, 300 mM KCl, 10 % glycerol (v/v), 1 mM DTT, 1 mM EDTA, 0.1 % Triton X-100 (v/v), 1 mM PMSF) and clarified by filtration to produce the Input. To 20 ml Input, 1 ml buffer A equilibrated IgG-Sepharose 6 Fast Flow (GE Healthcare, 17-0969-01) was added and rotated for 3 h at 4°C and washed with ice-cold buffer A. Telomerase was released from 1ml IgG-Sepharose by overnight incubation with 5 ml 0.05 mg/ml TEV protease (>90% efficient) in buffer A at 4°C .

Direct telomerase activity assay

Telomeric (TTAGGG)3 primer was incubated in reaction buffer with a super-telomerase extract [3], dTTP, dATP, dGTP and [α32-P]dGTP for 6 at 37 µC. Telomere DNA synthesis was analyzed on denaturing PAGE and the incorporation of [α32-P]dGTP was detected by radioisotope imaging. Alternatively dTTP was substituted with decreasing concentrations of ACV-TP-T. To determined the telomerase KMs for ACV-dT-T and dTTP telomerase activity was measured with the TeloSpot method [4]. TeloSpot involves incubation of super-

telomerase extracts for 45 min at 30ºC in 20 µl volumes reaction buffer containing 1 µl of, 50mM Tris-HCl (pH 8.0), 50mM KCl, 1mM spermidine, 1mM β mercaptoethanol, 1mM $MgCl_2$, of 0.25 µM telomeric $(TAGGG)_3$ primer, 1 mM dTTP, 1 mM dATP, 0.1 mM dGTP and 0.17 µM dGTP [α-32P] and where appropriate dTTP was substituted with 0.02 to 2 mM ACV-TP-dT. After overnight hybridization at 60 ºC with a randomly labeled probe derived from a 600 bp TTAGGG-repeat containing DNA fragment, membranes were washed twice for 15 min with 2x SSC buffer at room temperature and twice for 30 min with 2x SSC, 0.1 % SDS at 65 ºC and exposed to a phosphoimager screen. Spot intensities were quantified using 2D-densitometry and the Aida software (Raytest). To calculate the Km of the reaction the Lineweaver–Burk plot or double reciprocal plot was used; this is produced by taking the reciprocal of both sides of the Michaelis–Menten equation. This is a linear form of the Michaelis–Menten equation and produces a straight line with the equation $y = mx + c$ with a y-intercept equivalent to 1/Vmax and an x-intercept of the graph representing −1/KM.

Detection of pro-drug metabolites in HCC cells

Hepa1-6 cells ($5x10^6$ cells) were incubated with 1 mmol/L final concentration of ACV-TP-T. After 2-6 hours, cells were lysated with ice-cold 60% methanol-40% 15 mmol/L ammonium acetate buffer (pH 6.7). Cellular lysates were then transferred in YM-3 3000 MW cut-off Centricon tubes (Millipore, Milan, Italy) and centrifuged; the filtrated solutions were used for liquid chromatography tandem mass spectrometry analysis (LC-MS/MS). The unfiltrated samples were used for DNA extraction and the collected genomic materials underwent to extensive DNAse I digestion. The analysis was performed with LTQ linear ion trap mass spectrometer (Thermo Electronics, San Jose, CA, USA) directly coupled to the liquid chromatography through an electrospray interface. The liquid chromatography separation was performed using a Zic-Hilic 150 x 2.1 mm, 3.5 µm (Merck Sequant, Umea, Sweden) at a flow rate of 230 µL/min. The eluents were A 20 mmol/L ammonium acetate solution (pH

7.0) and B acetonitrile. The gradient applied was from 85% to 50% of B in 20 minutes, then back to the initial composition and maintained for 15 min for reconditioning. The analyses were performed in negative ion mode; for each analyte the deprotonated precursor ion was isolated and fragmented in the ion trap. The full product ion scan was recorded in the mass range from 80 to 400 m/z. For each analyte the signals of two principal fragment ions was extracted, then summed and the intensity recorded. The precursor ion and the two fragment ions of each analyte are listed in the following table:

Analyte	Precursor ion $(M-H)^-$	Fragment ions (collision energy)
ACV-P	304.0 m/z	152.8, 285.9 m/z (33%)
dTMP	321.1 m/z	176.9, 194.9 m/z (26%)
ACV-DP	384.0 m/z	365.9, 158.8 m/z (22%)
ACV-TP-T	688.0 m/z	256.8, 382.8 m/z (22%)

The concentration of ACV-TP-T in the cells was estimated by comparison of the peak area of a standard solution of ACV-TP-T with the peak area resulting from the analysis of cell lysate (the volume of $5x10^6$ Hepa1-6 cells required for calculating the concentration was estimated in 20 µL). No corrections were performed for matrix suppression/enhancement effect in the ESI interface.

Cell culture and transfection

HepG2, Huh7, Hep3B, Hepa 1-6 and 18CO were cultured in standard conditions in DMEM or EMEM (SIGMA-ALDRICH, Milan, Italy) with L-glutamine (2mM), 10% FBS and 1% Penicillin/Streptomycin solution (SIGMA-ALDRICH, Milan, Italy). Transient transfection of cell lines was performed with Lipofectamine 2000 reagent (Invitrogen, Monza, Italy) according to the manufacturer’s instructions; empty vector (pCMV), p53 expression vector

(pSN3-p53)[5], pBabe-puro, pBabe-puro-dominant negative human telomerase reverse transcriptase (hTERT-DN), pBabe-puro-human telomerase reverse transcriptase (hTERT-WT) or pBabe-puro-UThTERT+U3-hTR-500 (hTERT-hTR) (Addgene plasmids 1775, 1771 and 27665)[6-8] were used.

Telomerase activity assay in cell

In vitro telomerase activity was analysed using the telospot assay. Telospot telomerase activity assays were performed as previously described [9]. Cellular telomerase activity was tested by the TeloTAGGG Telomerase PCR ELISA PLUS kit (Roche, Milan, Italy). Briefly, 2 x 10^5 exponentially growing cells were lysed using the provided lysis buffer, and then the assay was carried out according to manufacturer's protocol.

Analysis of cell proliferation and viability

Cells were seeded in 96 well plates at a 15000 cells/well density; 24 h after seeding, the medium was changed and the pro-drugs were added for an additional 24 h. Cell viability was assessed by measuring the intracellular ATP content using CellTiter-Glo® Luminescent Cell Viability Assay (Promega, Milan, Italy). The experiment was run according to manufacturer's protocol, and luminescence was recorded with a Wallac multiplate reader (Perkin-Elmer, Monza, Italy). Cell proliferation was assessed by measuring radio-labeled thymidine incorporation. After seeding, cells were permitted to attach for 8 hours in complete medium, then media was changed to serum-free media with or without the pro-drugs. DNA synthesis in cell cultures was measured after incubation of cells with 5 µCi of [metyl-^{3}H]thymidine (85 Ci/mmol) for 24 hours followed by TCA precipitation.

Cell cycle analysis

For cell cycle analysis, single-cell suspensions were prepared and washed with cold PBS with 0.1% sodium azide. Cell pellets were gently resuspended in one part (0.3 ml) of 50% FBS in PBS. While gently mixing, three parts (0.9 ml) of cold 70% ethanol in distilled water were added drop-wise. Cells were then incubated for 2 hours at 4°C and then washed twice with cold PBS/sodium azide to remove ethanol and precipitated protein. Propidium iodide (PI) was added at 50 µg/ml in PBS containing 100 U/ml RNAase. Cells were then incubated for 30 minutes at room temperature. Data were analyzed on a BD LSRII cytofluorimeter (BD Biosciences, Milan, Italy) with the Modfit LT software (Verity Software House Inc., Topsham, Maine, USA).

Annexin V/propidium iodide staining

The evaluation of apoptosis was performed using the Annexin V kit (BD Biosciences, Milan, Italy) following the manufacturer's instructions. Apoptotic cells were identified by double supra-vital staining with recombinant APC-conjugated annexin V and PI. AnnexinV/PI analysis was performed on adherent cells plus detached cells present in the supernatant, and that cell doublets were excluded by the PE-A/PE-W method. Cells were then analyzed on a BD LSRII cytofluorimeter using the FACSDiva software. Cells that were Annexin V (-) and PI (-) were considered viable cells. Cells that were Annexin V (+) and PI (-) were considered early-stage apoptotic cells. Cells that were Annexin V (+) and PI (+) were considered late-stage apoptotic or secondary necrotic cells. Cells that were Annexin V (-) and PI (+) were considered dead cells, with stripped cytoplasmatic membranes leaving isolated nuclei or cellular debris.

Mouse models of Hepatocarcinogenesis

Tg[Alb-1 HBV]Bri 44 transgenic mice (Jackson Laboratories, Bar Harbor, ME, USA) express and accumulate in the hepatocytes the HBsAg protein, resulting in severe chronic

hepatocellular injury [10]. 15 months-old male mice (n = 40) were treated intraperitoneally with the ACV-TP T at the dose of 200 mg/kg (270 nmol/g) in 200 µL of saline solution 3 times a week for 4 weeks. Control mice received intraperitoneal injection of vehicle alone.

Four week old C57BL/6 male mice (n = 40) (HARLAN, Milan, Italy) were used to perform HCC implantation. Orthotopic implantation of cancer cells was performed as briefly described: Hepa1-6 cells were growth in complete medium until the cells reached 70% confluence when the medium was replace with fresh one to remove dead and detached cells; after 3 to 4 hours the added medium was removed and the cells were washed twice with PBS. A minimum amount of trypsin-EDTA was added. Cells were dispersed, complete medium was added (6:1) and immediately centrifuged at 1400 rpm for 2-5 min. Cells were counted with a hemocytometer, using trypan blue staining to exclude dead cells. 2.0×10^6 cells were suspended in a volume of 100 µl per injection. Animals were anesthetized with Avertin (240 mg/kg), 1 cm incision was done on the abdominal wall to expose the left lobe of the liver and the cells were injected with a 27G needle.. Animals recovered for 48h after the procedure before starting a 2 week treatment: 200 mg/kg/day (270 nmol/g) of ACV-TP-T in 100 µL in saline solution or vehicle alone was injected intraperitoneally. BAY 54-9085 (sorafenib tosylate) (Bayer HealthCare Pharmaceuticals, Montville, NJ, USA) 30 mg/kg/day or its vehicle (Cremophor/ ethanol/ distilled water) was administered by gavage. Animals were housed in temperature-, air-, and light- controlled conditions and received food and water *ad libitum.*

All animals received human care following institution guidelines.

Tumor evaluation

The size of all visible tumors was recorded and lesions were measured on the liver surface of the liver with a caliper at the time of sacrifice; the number and size of tumors were quantified. Hepatic tissues were fixed in 10% paraformaldehyde and embedded in paraffin.

Liver sections of 5µm were obtained from 4 different regions of the left lobe in the orthotopic model or of the whole liver in the transgenic HBV mice (at least 2 mm apart) [5]. Sections were stained with H&E, and the surface area was quantified (BIOQUANT NOVA PRIME Measurement Software, BIOQUANT Image Analysis Corporation, Nashville, TN, USA).

Immunohistochemistry (IHC)

Proliferation of hepatic cells was estimated by immunostaining for PCNA (Ab2426, Abcam, Cambridge, UK) whereas apoptosis was detected by staining for activated Caspase 3 (Ab13847, Abcam, Cambridge, UK). PCNA labeling index (LI) and apoptotic indices were semi-quantitatively evaluated by counting the percentage of immunoreactive cells in randomly selected high-power (X400) fields using the computerized video-image analysis system Quantimet Q500MC (Leica, Cambridge, UK).

Statistical analysis

Data are expressed as average ± SEM unless otherwise indicated. Student's t-test or one-way Anova were calculated to compare experiments. Differences were considered as statistically significant if $p<0.05$. Analyses were conducted using R statistical package (www.r-project.org) and SPSS® software (version 14).

Supplementary Figures

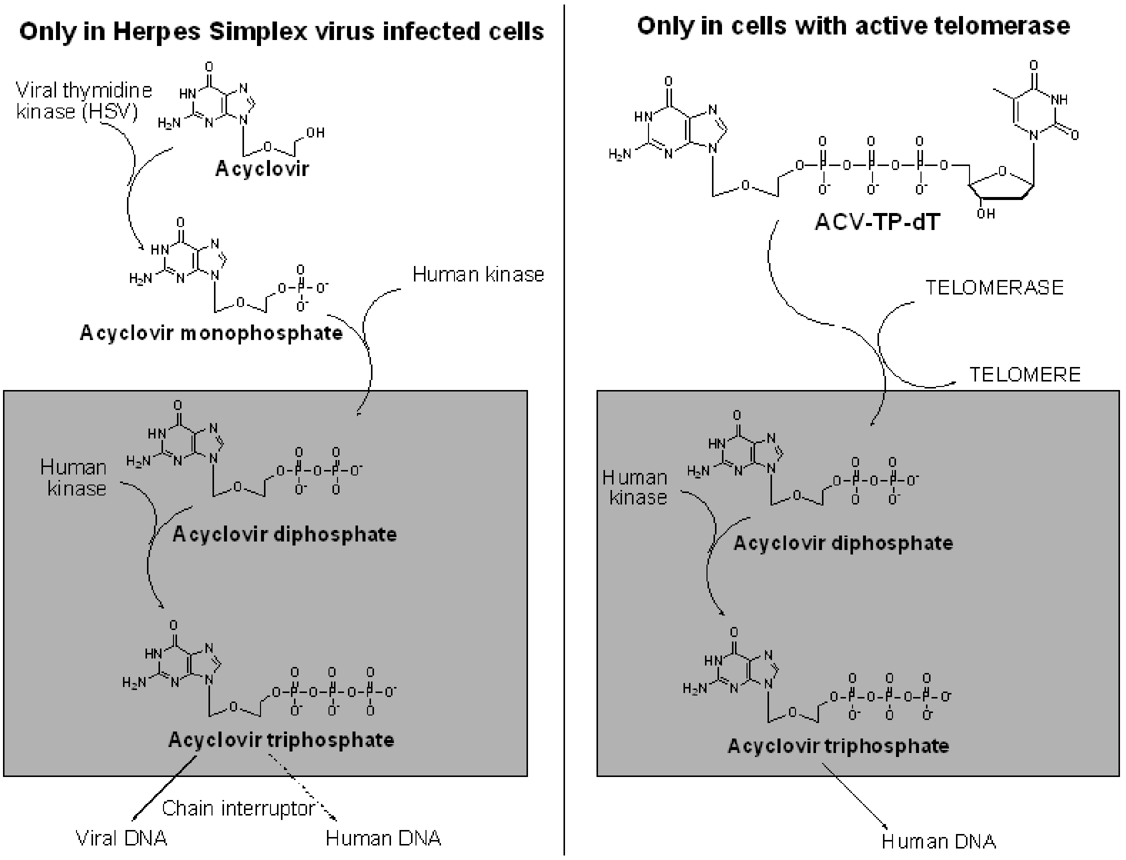

Supplementary Fig. 1. Prodrug activation mechanism. Structure and schematic mode of action of ACV-TP-T in comparison with ACV. For activation, ACV requires to be phosphorylated to ACV monophosphate by a viral TK carried by the herpes virus. Conversely, ACV-TP-T is a substrate of telomerase that incorporates the thymidine in the replicating telomeres and directly releases ACV diphosphate skipping the viral TK phosphorylation step.

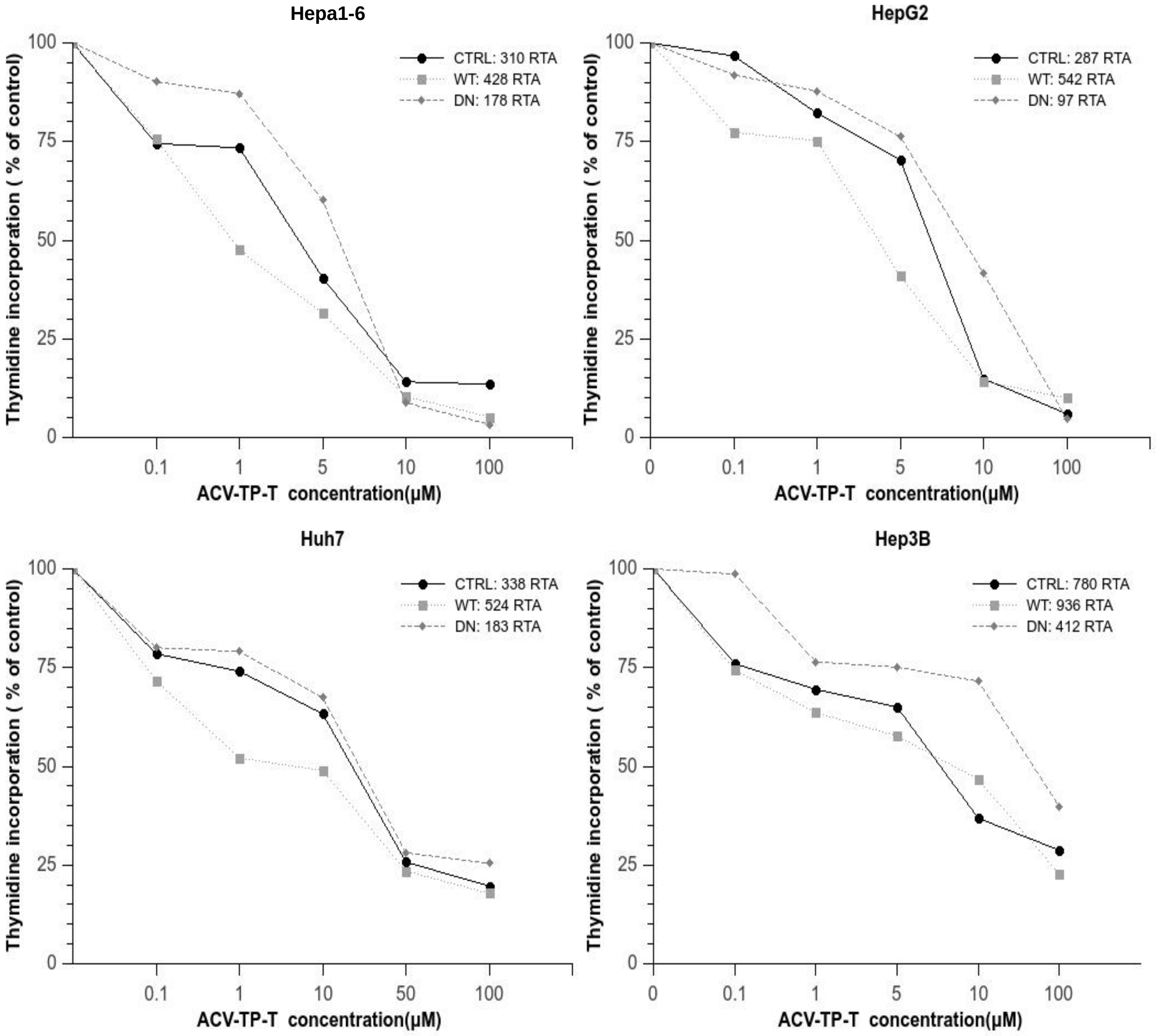

Supplementary Fig. 2. Modulation of hTERT expression in cancer cell lines alters sensitivity to ACV-TP-T. HepG2, Huh7, Hep3B and Hepa1-6 cells were transfected with WT-hTERT, DN-hTERT or empty vector (CTRL). 24 h after transfection, the cells were treated for 24 h with ACV-TP-T. Proliferation was tested by [^{3}H]thymidine incorporation. RTA, relative telomerase activity. Data showing one representative experiment out of three.

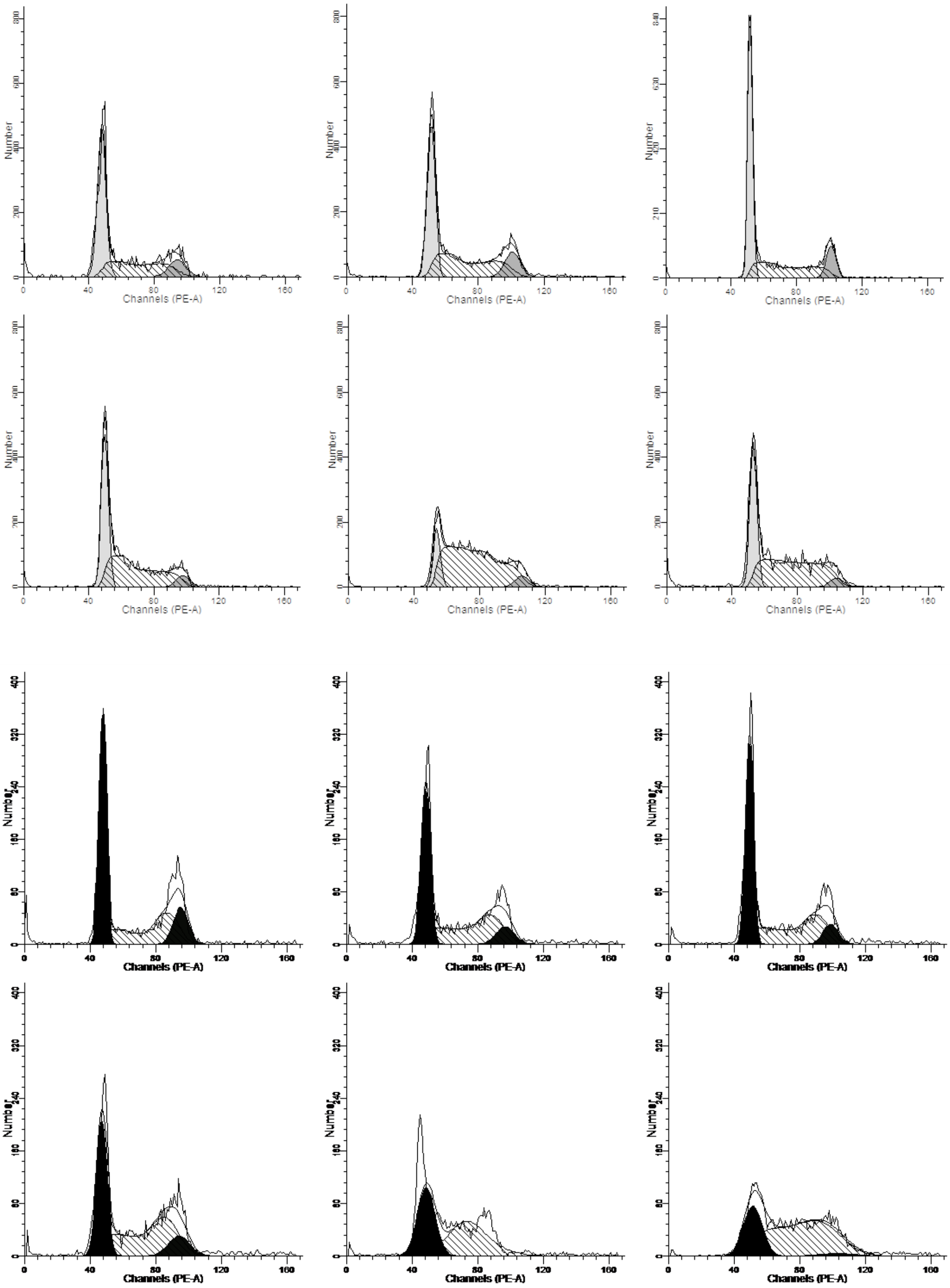

Supplementary Fig. 3. ACV-TP-T treatment blocks human cancer cells in S phase. (A) Cell cycle analysis of synchronized Huh7 cells treated with ACV-TP-T: data showing one representative experiment out of three with distribution of the cells in G0-G1, S phase, G2-M. (B) Cell cycle analysis of synchronized Hep3B cells treated with ACV-TP-T: data showing one representative experiment out of three with distribution of the cells in G0-G1, S phase, G2-M.

A

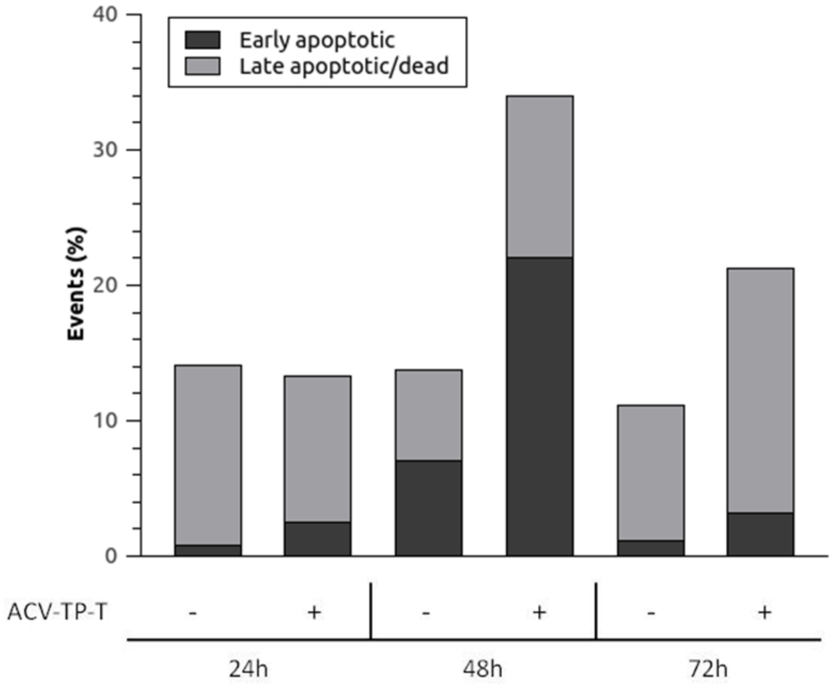

B

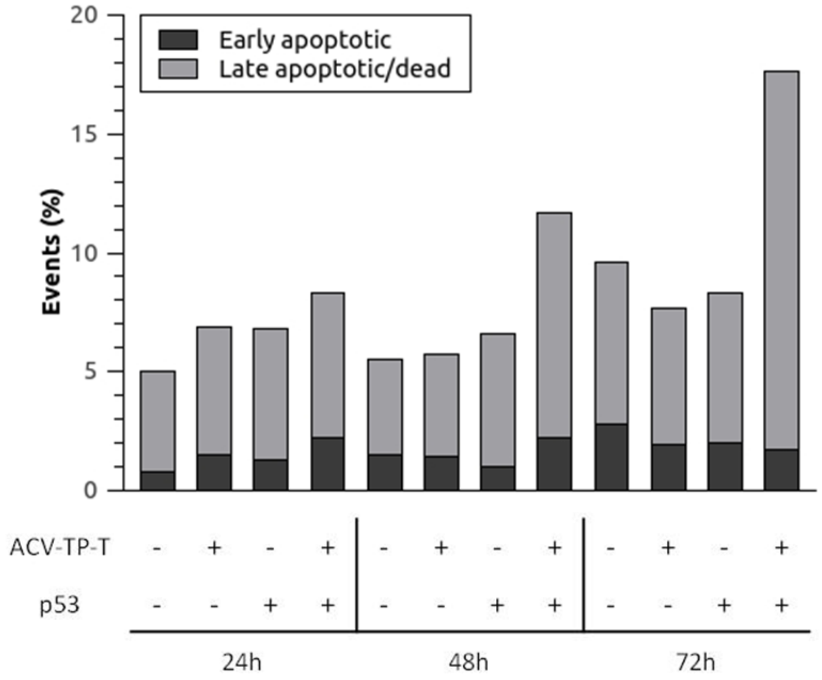

Supplementary Fig. 4. ACV-TP-T treatment increases human cancer cell death. (A) Flow cytometry analysis of Huh7 cell vitality at 24, 48 and 96 hours (one representative experiment out of three): apoptotic and dead cells are expressed as % of total events. (B) Flow cytometry analysis of Hep3B cell vitality at 24, 48 and 96 hours with or without the expression of wild type p53 (one representative experiment out of three): apoptotic and dead cells are expressed as % of total events.

A

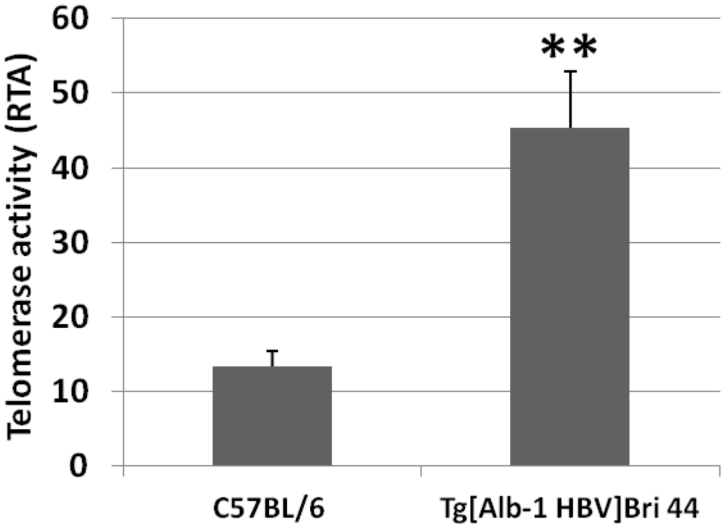

B

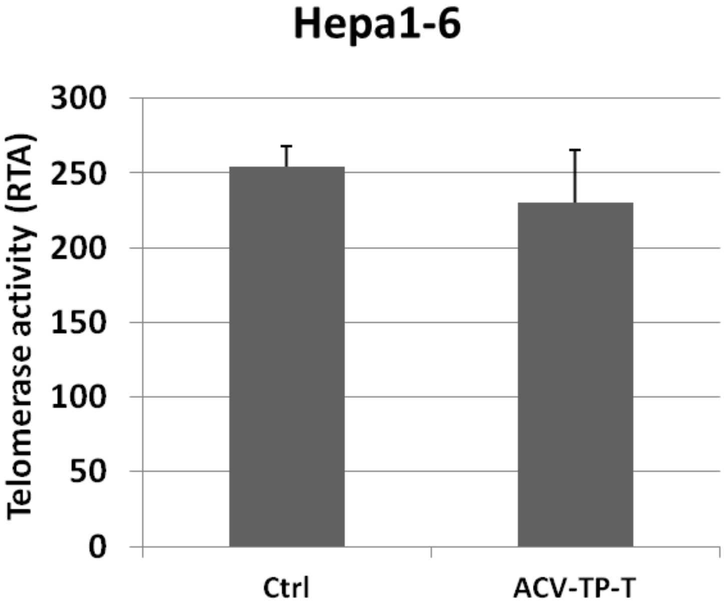

Supplementary Fig. 5. Telomerase reactivation in transgenic mice. Telomerase activity was analyzed by the TeloTAGGG Telomerase PCR ELISA PLUS kit. (A) RTA was tested according to manufacturer's protocol in liver tissues from 15 month old Tg[Alb-1 HBV]Bri 44 transgenic mice (n = 3) and compare to wild type animals (n = 3). (expressed as average ± SD, ***p* <0.01). (B) RTA was tested according to manufacturer's protocol in $2x10^5$ Hepa1-6 after 24h treatment with ACV-TP-T (expressed as average ± SD).

References

[1] Cristofari G, Adolf E, Reichenbach P, Sikora K, Terns R, Terns M, et al. Human telomerase RNA accumulation in Cajal bodies facilitates telomerase recruitment to telomeres and telomere elongation. Mol Cell 2007;27:882-889.

[2] Cohen S, Graham M, Lovrecz G, Bache N, Robinson P, Reddel R. Protein composition of catalytically active human telomerase from immortal cells. Science 2007;315:1850-1853.

[3] Cristofari G, Lingner J. Telomere length homeostasis requires that telomerase levels are limiting. EMBO J 2006;25:565-574.

[4] Cristofari G, Reichenbach P, Regamey PO, Banfi D, Chambon M, Turcatti G, et al. Low- to high-throughput analysis of telomerase modulators with Telospot. Nat Methods 2007;4:851-853.

[5] Tarocchi M, Hannivoort R, Hoshida Y, Lee UE, Vetter D, Narla G, et al. Carcinogen-induced hepatic tumors in KLF6+/- mice recapitulate aggressive human hepatocellular carcinoma associated with p53 pathway deregulation. Hepatology 2011;54:522-531.

[6] Counter CM, Hahn WC, Wei W, Caddle SD, Beijersbergen RL, Lansdorp PM, et al. Dissociation among in vitro telomerase activity, telomere maintenance, and cellular immortalization. Proc Natl Acad Sci U S A 1998;95:14723-14728.

[7] Hahn WC, Stewart SA, Brooks MW, York SG, Eaton E, Kurachi A, et al. Inhibition of telomerase limits the growth of human cancer cells. Nat Med 1999;5:1164-1170.

[8] Wong JM, Collins K. Telomerase RNA level limits telomere maintenance in X-linked dyskeratosis congenita. Genes Dev 2006;20:2848-2858.

[9] Cristofari G, Reichenbach P, Regamey P, Banfi D, Chambon M, Turcatti G, et al. Low- to high-throughput analysis of telomerase modulators with Telospot. Nat Methods 2007;4:851-853.

[10] Galli A, Ceni E, Mello T, Polvani S, Tarocchi M, Buccoliero F, et al. Thiazolidinediones inhibit hepatocarcinogenesis in hepatitis B virus-transgenic mice by peroxisome proliferator-activated receptor gamma-independent regulation of nucleophosmin. Hepatology 2010;52:493-505.
